# Endothelial ether lipids link the vasculature to blood pressure, behavior, and neurodegeneration

**DOI:** 10.1016/j.jlr.2021.100079

**Published:** 2021-04-21

**Authors:** Larry D. Spears, Sangeeta Adak, Guifang Dong, Xiaochao Wei, George Spyropoulos, Qiang Zhang, Li Yin, Chu Feng, Donghua Hu, Irfan J. Lodhi, Fong-Fu Hsu, Rithwick Rajagopal, Kevin K. Noguchi, Carmen M. Halabi, Lindsey Brier, Annie R. Bice, Brian V. Lananna, Erik S. Musiek, Oshri Avraham, Valeria Cavalli, Jerrah K. Holth, David M. Holtzman, David F. Wozniak, Joseph P. Culver, Clay F. Semenkovich

**Affiliations:** 1 Division of Endocrinology, Metabolism & Lipid Research, Department of Medicine, Washington University, St. Louis, MO, USA; 2 Hubei Key Laboratory of Animal Nutrition and Feed Science, Wuhan Polytechnic University, Wuhan, China; 3 Department of Pediatrics, Washington University, St. Louis, MO, USA; 4 Department of Ophthalmology & Visual Sciences, Washington University, St. Louis, MO, USA; 5 Department of Psychiatry, Washington University, St. Louis, MO, USA; 6 Department of Radiology, Washington University, St. Louis, MO, USA; 7 Department of Neurology, Washington University, St. Louis, MO, USA; 8 Department of Neuroscience, Washington University, St. Louis, MO, USA; 9 Department of Cell Biology & Physiology, Washington University, St. Louis, MO, USA

**Keywords:** glycerophospholipids, plasmalogens, phospholipids/biosynthesis, peroxisomes, vascular biology/endothelial cells, GFAP, glial fibrillary acidic protein, GSK3, glycogen synthase kinase-3, HR, high resolution, LC, locus coeruleus, LIT, linear ion trap, MS^n^, multiple stage mass spectrometry, PC, phosphatidylcholine, PE, phosphatidylethanolamine, PEKO, PexRAP endothelial knockout, PMKO, PexRAP muscle knockout

## Abstract

Vascular disease contributes to neurodegeneration, which is associated with decreased blood pressure in older humans. Plasmalogens, ether phospholipids produced by peroxisomes, are decreased in Alzheimer's disease, Parkinson's disease, and other neurodegenerative disorders. However, the mechanistic links between ether phospholipids, blood pressure, and neurodegeneration are not fully understood. Here, we show that endothelium-derived ether phospholipids affect blood pressure, behavior, and neurodegeneration in mice. In young adult mice, inducible endothelial-specific disruption of PexRAP, a peroxisomal enzyme required for ether lipid synthesis, unexpectedly decreased circulating plasmalogens. PexRAP endothelial knockout (PEKO) mice responded normally to hindlimb ischemia but had lower blood pressure and increased plasma renin activity. In PEKO as compared with control mice, tyrosine hydroxylase was decreased in the locus coeruleus, which maintains blood pressure and arousal. PEKO mice moved less, slept more, and had impaired attention to and recall of environmental events as well as mild spatial memory deficits. In PEKO hippocampus, gliosis was increased, and a plasmalogen associated with memory was decreased. Despite lower blood pressure, PEKO mice had generally normal homotopic functional connectivity by optical neuroimaging of the cerebral cortex. Decreased glycogen synthase kinase-3 phosphorylation, a marker of neurodegeneration, was detected in PEKO cerebral cortex. In a co-culture system, PexRAP knockdown in brain endothelial cells decreased glycogen synthase kinase-3 phosphorylation in co-cultured astrocytes that was rescued by incubation with the ether lipid alkylglycerol. Taken together, our findings suggest that endothelium-derived ether lipids mediate several biological processes and may also confer neuroprotection in mice.

Neurodegenerative disorders commonly occur in the setting of vascular disease (1). Cerebrovascular lesions spanning a spectrum of atherosclerosis and hypoperfusion coexist with the neuropathology of Alzheimer's disease, Parkinson's disease, and other causes of dementia (2, 3, 4). Known mediators of vascular disease such as hypertension, diabetes, and hyperlipidemia in midlife increase the risk of dementia in late life (5). Cardiovascular-associated genes in addition to APOE are related to dementia (6, 7), consistent with the notion that vascular dysfunction, perhaps exacerbated by amyloid β (8) and tau (9), promotes neurodegeneration.

Vascular dysfunction is associated with abnormalities of blood pressure, but the relationship between blood pressure and dementia is not straightforward. While midlife hypertension increases dementia risk in old age, there is little evidence that antihypertensive therapy prevents neurodegeneration when started in later life (10, 11). Most individuals with dementia have low blood pressure (12, 13, 14), the pattern of midlife hypertension and late-life hypotension is associated with dementia (15), and a decrease in blood pressure often precedes the development of dementia (16) through unknown mechanisms.

Complex phospholipids could provide a mechanistic connection between neurodegeneration and the vasculature. Plasmalogens are the most abundant form of ether phospholipids, which require peroxisomes for synthesis. Both the central and peripheral nervous systems are enriched in plasmalogens that are critical components of synaptic vesicles and myelin where they are implicated in signaling events and protection from oxidation (17, 18).

Several lines of evidence connect ether lipids and neurodegeneration. Plasmalogens decrease with age in the serum and brain (19, 20). Loss of function mutations in human peroxisomal genes cause several types of rhizomelic chondrodysplasia punctata, a fatal developmental disorder characterized by low levels of plasmalogens and severe neurologic deficits (18). Mouse models of ether lipid deficiency show neurodegeneration (18). Ethanolamine plasmalogens, the dominant form of ether lipids in neural tissue, are decreased in the brain and circulation of patients with Alzheimer's disease (21, 22, 23, 24, 25), Parkinson's disease (26, 27), and Down syndrome (28, 29).

Decreased plasmalogens could be a consequence of the neurodegenerative process, but circulating phospholipids are reported to predict dementia (24, 30), raising the possibility that alterations in lipid species generated at sites distinct from neuronal tissue could contribute to or be a cause of neurodegeneration. Since the endothelium comprises the blood-brain barrier and is altered in hypertension, we inactivated PexRAP, an enzyme required for ether lipid synthesis, in endothelial cells of young adult mice to test the hypothesis that vascular plasmalogen generation impacts neurodegeneration.

## Materials and Methods

### Animals

PexRAP endothelial knockout (PEKO) mice were generated by mating PexRAP^lox/lox^ mice on a C57BL/6J genetic background (31) with VE-cadherin-Cre-ERT2 mice (Tg(Cdh5-cre/ERT2)1Rha). Endothelial PexRAP deletion was induced by injecting tamoxifen (75 mg tamoxifen/kg body weight i.p.) for five consecutive days beginning at ∼35 days of age. Littermate tamoxifen-injected PexRAP^lox/lox^ mice without Cre were controls. PexRAP muscle knockout (PMKO) mice were generated by mating PexRAP^lox/lox^ mice with transgenic mice expressing Cre driven by the human α-skeletal actin promoter. PexRAP heterozygote (*Dhrs7b+/-*) mice were produced by Lexicon Pharmaceuticals (The Woodlands, TX) and Genentech (South San Francisco, CA) (32) and obtained by Dr Alexander Moise through the NIH Mutant Mouse Regional Resource Center. Mice were fed commercial chow, or an 8% high-salt diet (Envigo TD.03142). Similar numbers of male and female mice were used in all experiments. All protocols were approved by the Washington University Animal Studies Committee and followed the guidelines of the NIH Guide for the Care and Use of Laboratory Animals.

### Mass spectrometry

For plasmalogens, samples were normalized by protein content, and lipids were extracted according to the Bligh & Dyer method (33) in the presence of the internal standards: 14:0-14:0 PC ([M+H]^+^ *m/z* 678.51) and 14:0-14:0 PE([M-H]^-^ m/z 634.45) from Avanti. While these species are not typically found in mice, control studies were performed in PEKO mice to verify that the 14:0, 14:0 species were not detectable in animals with endothelial deficiency of PexRAP (supplemental Fig. S1). After 1 min of vortexing and 5 min of centrifugation (800 *g*), the lower organic layer of the extract was collected. The organic layer was dried under nitrogen gas and reconstituted in methanol with 0.25% NH_4_OH and loop injected into the electrospray ion source. A Thermo Fisher Vantage triple-quadruple mass spectrometer operated by Xcalibur software was used for semiquantitation of phosphatidylcholine (PC) species in the positive ion mode using precursor ion scan of 184 with collision energy of 32 eV and of phosphatidylethanolamine (PE) species in the negative ion mode using precursor ion scan of 196 with collision energy of 55 eV (34). Semiquantification was carried out by comparing species intensities against the intensity of the added internal standard.

**Fig. 1:**
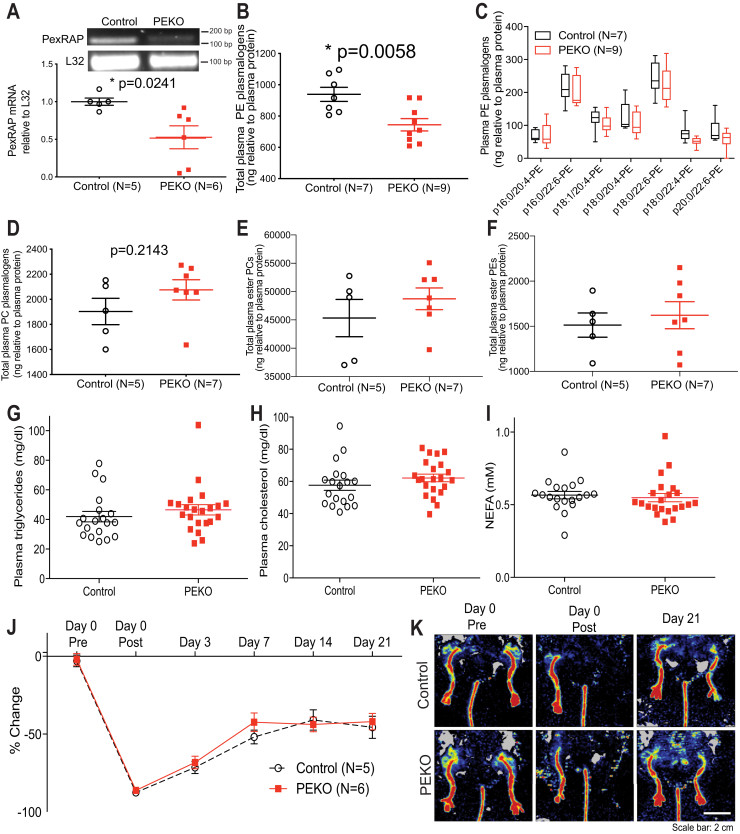
PEKO (PexRap Endothelial KnockOut) mice have decreased circulating PE plasmalogens, but normal concentrations of other lipids and a normal response to hindlimb ischemia. A: QRT-PCR of PexRAP mRNA in lung endothelial cells from control and PEKO mice with representative images of PexRAP and L32 amplification products above graphs. B: Total PE plasmalogens determined by mass spectrometry in plasma from control and PEKO mice. C: Individual PE plasmalogen species in plasma from control and PEKO mice. D: Total PC plasmalogens, (E) total ester phosphatidylcholine species, and (F) total ester phosphatidylethanolamine species in plasma from control and PEKO mice. G–I: Fasting triglycerides (G), cholesterol (H), and nonesterified fatty acids (NEFA, I) in plasma from control and PEKO mice. J: Angiogenesis responses following hindlimb ischemia in control and PEKO mice. K: Representative laser Doppler imaging of mice before and after femoral artery ligation. Data are presented as individual data points with mean ± SEM, box and whisker plots, or mean ± SEM over time. PE, phosphatidylethanolamine.

For confirmation and distinction of plasmalogen and 1-O-alkyl 2-acyl PE and PC species, lipid extracts were subjected to high resolution (HR) (R = 100,000 at m/z 400) linear ion trap (LIT) multiple stage mass spectrometry (MS^n^) using a Thermo Fisher LTQ orbitrap Velos, operated by Xcalibur software as previously described (35, 36). Samples were loop injected or continuously infused into the high-temperature electrospray ionization source, where the ionization voltage was set at 4.5 kV, and the temperature of the heated capillary was 300°C. The automatic gain control of the ion trap was set to 5 × 10^4^, with a maximum injection time of 200 ms. Helium was used as the buffer and collision gas at a pressure of 1 × 10^−3^ mbar (0.75 mTorr). The MS^n^ experiments were carried out with an optimized relative collision energy ranging from 30% to 40% and with an activation q value at 0.25 and the activation time at 10 ms. Mass spectra were accumulated in the profile mode, typically for 3–10 min for MS^n^ (n = 2, 3, 4) spectra. The combined results from HR LIT MS^n^ demonstrated that the [M – H]^−^ ion of ether PE species of m/z 750.5445, for example, corresponds to an elemental composition of C_43_H_77_O_7_NP (calculated m/z:750.5443). The MS^2^ spectrum of m/z 750, along with MS^3^ spectrum of m/z 464 (750 → 464) that contained an abundant ion of 267.2687 (C_18_H_35_O^−^; calc m/z 267.2593) indicating the presence of p18:0, led to the assignment of p18:0/24:4-PE structure, in addition to the minor p16:0/22:4-PE isomer. Similar HR LIT MS^n^ approaches were applied to define the other plasmalogen PE structures as shown in Fig. 1C.

For proteins, samples were solubilized, reduced, alkylated, trypsinized, washed, acidified, and desalted before being subjected to LC-ESI/MS/MS. Data were acquired using a Thermo Fisher Q-Exactive™ Plus Hybrid Quadrupole-Orbitrap™ Plus mass spectrometer (Thermo Fisher Scientific) coupled to an EASY-nanoLC 1000 system. Full-scan mass spectra were acquired by the Orbitrap™ mass analyzer in the mass-to-charge ratio (*m/z*) of 375–1,500 and with a mass resolving power set to 70,000. Twelve data-dependent high-energy collisional dissociations were performed with a mass resolving power set to 17,500, an isolation width of 1.2 Da, and a normalized collision energy setting of 27. The maximum injection time was 60 ms for both parent-ion analysis and product-ion analysis. The target ions that were selected for MS/MS were dynamically excluded for 20 s. The automatic gain control were set at a target value of 1e6 ions for full MS scans and 1e5 ions for MS2. Peptide ions with charge states of 1 or > 6 were excluded for collision induced dissociation acquisition.

Protein identifications were generated at the Washington University Proteomics Shared Resource. MS raw data were converted to peak lists using Proteome Discoverer (version 2.1.0.81, Thermo Fischer Scientific). MS/MS spectra with charges +2, +3, and +4 were analyzed using Mascot search engine (Matrix Science, London, UK; version 2.5.1). Mascot was set up to search against a UniRef database of mouse (version October, 2013; 43,296 entries) and common contaminant proteins (cRAP, version 1.0 January 1, 2012; 116 entries), assuming the digestion enzyme was trypsin/P with a maximum of four missed cleavages allowed. The searches were performed with a fragment ion mass tolerance of 0.02 Da and a parent ion tolerance of 20 ppm. Carbamidomethylation of cysteine was specified in Mascot as a fixed modification. Deamidation of asparagine, formation of pyro-glutamic acid from N-terminal glutamine, acetylation of protein N-terminus, oxidation of methionine, and pyro-carbamidomethylation of N-terminal cysteine were specified as variable modifications. Peptide spectrum matches were filtered at 1% false discovery rate by searching against a reversed database, and the ascribed peptide identities were accepted. The uniqueness of peptide sequences among the database entries was determined using the principal of parsimony, and protein identities containing > 2 unique peptides were accepted, followed by spectral counting quantifications against the protein data.

### Blood pressure determinations

Blood pressure was measured by using a tail cuff device and implantable transmitters. Systolic blood pressure, diastolic blood pressure, and heart rate were measured in conscious, acclimated mice using a tail-cuff system (Kent Scientific). Multiple measurements were performed over 3 to 5 days and averaged. Investigators were blinded to genotype for these initial studies. For salt-loading experiments, multiple measurements were performed on the same day. For telemetry, mice were anesthetized, given an analgesic before surgery, and prepared according to AAALAC-recommended aseptic techniques. Placement of telemetric devices (model PAC10, Data Sciences International) included inserting the telemetry catheter in the left carotid artery and placing the transmitter in a subcutaneous pocket at the abdomen. Tissue adhesive was used to secure the transmitter and stabilize the catheter. The wound was closed with sterile sutures that were removed 1 week later. Mice were kept on a heating pad and monitored closely until fully recovered from anesthesia. Blood pressure and heart rate recordings were initiated after suture removal and surgical recovery. Data were collected using RPC1 receivers that were activated by a magnetic device briefly positioned close to the animal from outside the cage. Investigators were not blinded to genotype for the salt-loading and telemetry analyses.

### Phenotyping and behavioral analyses

Chemistries in fasted mice and forelimb muscle strength using a Rodent Grip Strength Meter were measured as described (37). To measure angiogenesis after hindlimb ischemia, the femoral artery was ligated and transected as described (38). Perfusion was quantified by laser Doppler imaging for 21 days. Movements in the X+Y and Z axes were quantified by laser beam breaks in acclimated animals housed in TSE Phenomaster Metabolic Cages. Electroretinography in dark-adapted and light-adapted mice was performed using a UTAS BigShot System (39). Sleep was determined using a noninvasive automated sleep/wake system (PiezoSleep, Signal Solutions). Two months after treatment with tamoxifen, mice were acclimated to cages fitted with piezoelectric sensors. Data were collected over 3 days and analyzed using the Sleep Statistics Toolbox. One hour locomotor activity, novel object recognition, hanging object recognition, Morris Water Maze, inclined screen, and conditional fear testing were performed as described (40, 41) in the Washington University Animal Behavior Core. Investigators were blinded to genotype for these neurologic phenotyping studies.

### Histology

For all analyses, observers were blinded to genotype. Animals were deeply anesthetized and perfused via the left ventricle with 4% paraformaldehyde in 0.1 M Tris buffer. Brains were postfixed for 7 days before weighing and coronal sectioning at 75 μm on a vibratome. For tyrosine hydroxylase labeling, serial sections were quenched using methanol with 3% hydrogen peroxide for 10 min, blocked using 2% BSA, 0.2% milk, 0.1% Triton-X in PBS for an hour, and incubated overnight in 1:1,000 anti-tyrosine hydroxylase (1:500, MilliporeSigma). Sections were incubated in goat anti-rabbit secondary antibody (Vector Laboratories), reacted with Vectastain ABC Elite Kit, and visualized using the Vector VIP chromogen reagent. For Nissl staining, sections were mounted and dried on coated slides overnight, then incubated in a 0.1% cresyl violet/0.5% glacial acetic acid solution for 10 min. Sections were rinsed in distilled water and differentiated in 95% ethanol before dehydrating in a series of alcohols and CitriSolv. Silver staining, a marker of recent neurodegeneration, was performed as described (42).

For hippocampal glial fibrillary acidic protein (GFAP), mice were anesthetized by i.p. pentobarbital (150 mg/kg), followed by perfusion for 3 min with ice-cold Dulbecco's modified PBS containing 3 g/l heparin. The brain was extracted and postfixed in 4% paraformaldehyde for 24 h (4°C), then cryoprotected with 30% sucrose in PBS (4°C) for 48 h. Fifty micron serial coronal sections were then cut on a freezing sliding microtome and stored in cryoprotectant solution (30% ethylene glycol, 15% sucrose, 15% phosphate buffer). Sections were washed in TBS, blocked for 30 min in TBSX (TBS with 0.25% Triton X-100) containing 3% goat serum, then incubated overnight in TBSX containing 1% goat serum plus GFAP antibody (Dako/Agilent Z0334) at 4°C. Sections were then washed in TBS, incubated for 1 h at room temperature in TBSX with 1:1,000 fluorescent secondary antibody, and mounted on slides using Fluoromount G. 10x Epiflourescent images were obtained on a Nikon Eclipse 80i fluorescent microscope. GFAP staining was quantified from 10x images using ImageJ software. Hippocampi were outlined in GFAP 8 bit images, which were thresholded by hand to encompass all GFAP immunoreactivity. A threshold value was set and maintained for all sections. GFAP area coverage was then calculated for each outlined hippocampus. Two images (one/hippocampus) from each section and two sections/mouse were averaged, and the average from each mouse used as a data point.

For dorsal root ganglia GFAP, dorsal root ganglia were fixed by perfusion with PBS followed by 4% paraformaldehyde, isolated, and immersed in 4% paraformaldehyde. Tissue was cryoprotected in 30% sucrose in PBS, and 10 μm sections were generated. Slides were blocked in PBS with 0.1% Triton X-100 and 10% donkey serum. Sections were incubated overnight at 4°C in primary antibodies diluted in blocking reagent: GFAP (1:500; Thermo Fisher Scientific #18-0063) and chicken anti-βIII tubulin (TuJ1) (1:1,000; Abcam #ab41489). Tissue was washed several times, incubated with fluorescent-conjugated secondary antibodies (1:500; Thermo Fisher) and DAPI (1:1,000) diluted in blocking reagent, washed, and mounted in ProLong Gold antifade mountant (Thermo Fisher). Images were taken with a Nikon TE-2000E compound microscope and analyzed using NIS-Elements software (Nikon).

### Functional connectivity

Surgery consisted of scalp retraction and fixation of a plexiglass head plate with translucent dental cement on top of a nonthinned, fully intact skull (43) allowing for repeated imaging. Animals were allowed a week recovery period before data acquisition.

Optical intrinsic signal imaging was performed on anesthetized (86.9 mg/kg ketamine and 13.4 mg/kg xylazine) WT and PEKO mice, before and after tamoxifen injection. As described (44), the optical intrinsic signal apparatus provided sequential LED illumination (478 nm, 588 nm, 610 nm, 625 nm) of the mouse cortex, and light reflectance was captured by a high powered, cooled, frame-transfer EMCCD camera (iXon 897, Andor Technologies) at 120 Hz (30 Hz per LED). The 1 cm^2^ field-of-view captured the cortex spanning from the olfactory bulb to the superior colliculus. Data were binned into 128 × 128 pixels with a spatial resolution of approximately 78 μm^2^ per pixel. Offline, the light reflectance data were filtered to 0.009–0.08 Hz and translated to concentrations of oxygenated and deoxygenated hemoglobin by solving the modified Beer Lambert law. A binary brain mask was created for each mouse to discard pixels with specular reflections corresponding to nonbrain regions. The global signal within the brain region was averaged and regressed from the hemoglobin time traces. Zero-lag functional connectivity analysis was performed using predetermined “seeds” corresponding to left and right olfactory, frontal, cingulate, motor, somatosensory, retrosplenial, and visual cortices.

### Endothelial-astrocyte co-culture

bEnd.3 and C8-D1A [Astrocyte type I clone] cells (passage 15–30) from ATCC were cultured in DMEM with 10% FBS, 2 mM L-glutamine and 1% penicillin–streptomycin. bEnd.3 cells were seeded on the albuminal side of Transwell filters (0.4 μm pore size, 6-well) at 1 × 10^5^ cells per filter, allowed to adhere for 2 h, then filters were inverted in media.

For PexRap knockdown in bEnd.3 cells, 293T cells were transfected with packaging vectors, and an expression plasmid (pLKO.1-puro system) containing shRNA sequences that were scrambled or specific for mouse PexRap. Viruses were collected and filtered 2 days later, then used to infect bEnd.3 cells that were selected with puromycin for 2 days. After selection, the inserts with bEnd.3 cells were transferred to a six-well dish seeded with 1 × 10^5^ cells C8-D1A astrocyte-like cells per well and co-cultured for 24 h. bEnd.3 cells were assayed for PexRAP expression. Astrocyte-like cells were subjected to proteomic analysis by mass spectrometry and phospho-glycogen synthase kinase-3 (GSK3) assays by ELISA (R&D Systems). For rescue experiments, 20 μM of 1-O-hexadecyl-rac-glycerol (16:0 alkylglycerol) and 1-O-octadecyl-rac-glycerol (18:0 alkylglycerol) or vehicle (ethanol) was included in the co-culture system and incubated for 24 h.

### Quantitative RT-PCR and Western blotting

Total RNA was extracted with TRIZOL (Invitrogen) and reverse transcribed using an iScript cDNA synthesis kit (Bio-Rad). PCR reactions were performed with primer sequences as described (31). Protein extracts from mouse cortex were blotted to nitrocellulose and incubated with the following primary antibodies: Phospho-GSK-3α/β (Ser21/9) #9331 and GSK-33α/β #5676 (Cell Signaling).

### Statistics

Results are expressed as box and whisker plots, individual data points, or mean ± SEM. Most comparisons were performed by unpaired two-tailed *t* test or two-way ANOVA. Behavioral data were analyzed using ANOVA models, including repeated measures ANOVAs to evaluate data containing a time dimension or test trials. The repeated measures ANOVA models typically included two between-subjects variables (genotype and sex) and one within-subjects variable (e.g., time or blocks of trials). Simple main effects were calculated in the case of a significant interaction, and *P*-values were corrected using the Huynh-Feldt method to help protect against sphericity violations. Planned comparisons were conducted, and significance corrected for multiple comparisons using the Bonferroni method. Functional connectivity matrices were computed by calculating the Pearson correlation coefficient between two seed regions. Bilateral functional connectivity analysis was performed by isolating each brain pixel and its homotopic pixel and calculating the Pearson correlation coefficient between the two time traces. A Fisher z-transformation was used to perform mathematical operations on all Pearson correlation coefficients. Cross cortex correlation differences between controls and PEKO mice were tested using an independent two-tailed *t* test assuming unequal variances (Welch's *t* test).

## Results

### Induction of endothelial-specific deficiency of the ether lipid-generating enzyme PexRAP affects circulating plasmalogens

Floxed PexRAP (encoded by *Dhrs7b*) mice on a C57BL/6J background (31) were crossed with mice carrying an endothelial-specific inducible Cre, VE-Cadherin-CreERT2 (45). Floxed and floxed/Cre mice in the absence of tamoxifen were phenotypically normal. At ∼35 days of age, males and females were treated with tamoxifen to generate PEKO mice (supplemental Fig. S2A–D) that were studied between the ages of 3 and 6 months. Littermate control mice bearing floxed PexRAP alleles without Cre were also treated with tamoxifen. PexRAP message was decreased in the lung (a tissue enriched in endothelial cells) of PEKO as compared with control mice (Fig. 1A). PexRAP message was also decreased in endothelial cells isolated from the lung as compared with other cells (supplemental Fig. S2D). There was no effect of endothelial PexRAP inactivation on body weight or food intake as compared with controls.

PexRAP synthesizes ether lipids in peroxisomes, which are found in all mammalian tissues (46). Circulating plasmalogens reside in lipoproteins after synthesis in the liver (47). Loss of ether lipid production by the single layer of endothelial cells lining the vasculature would not be predicted to impact circulating lipid abundance, but plasma PE plasmalogens were unexpectedly decreased in PEKO mice compared to controls (Fig. 1B, C). Plasma PC plasmalogens were unaffected (totals Fig. 1D, individual species supplemental Fig. S3A). PexRAP can synthesize ester lipids as well as ether lipids (48). However, plasma ester PCs (totals Fig. 1E, individual species supplemental Fig. S3B) and plasma ester PEs (total Fig. 1F, individual species supplemental Fig. S3C) were unaffected. There was no effect of endothelial PexRAP deficiency on plasma triglycerides, cholesterol, or fatty acids (Fig. 1G–I).

Plasmalogens are alkenyl ether lipids, with a cis double bond adjacent to the ether bond at the sn-1 position. Alkyl ether lipids, which do not carry this double bond adjacent to the ether bond, were also quantified in plasma. There was no difference in the plasma content of alkyl ether PCs in control and PEKO mice (supplemental Fig. S3D for total content, supplemental Fig. S3E for individual species). We were unable to detect alkyl ether PEs in either genotype. For PCs, the semiquantitative distribution of diradyl glycerophospholipids was 96% diacyl, 4% 1-O-alkenyl-2-acyl, and 0.2% 1-O-alkyl-2-acyl. PC plasmalogens were more abundant than PE plasmalogens (Fig. 1D vs. Fig. 1B), and diacyl PCs were more abundant than diacyl PEs (Fig. 1E vs. Fig. 1F) We did not detect any platelet activating factors under these conditions. A series of ions with the same elemental composition as platelet activating factors were detected, but tandem mass spec of one of these sodiated species revealed a spectrum identical to that obtained using lyso-16:0-PC standard, indicating that these ions are lysophosphatidylcholines. Sphingomyelin and phosphatidylinositol content did not differ between control and PEKO mice (supplemental Fig. S3F, G).

Endothelial dysfunction is characterized by decreased bioavailable nitric oxide, which elevates blood pressure (49) and impairs angiogenesis following injury (50). PEKO mice had the same angiogenic response as controls in hindlimb ischemia experiments (Fig. 1J, K).

### PEKO mice have altered blood pressure and behavior

**Fig. 2:**
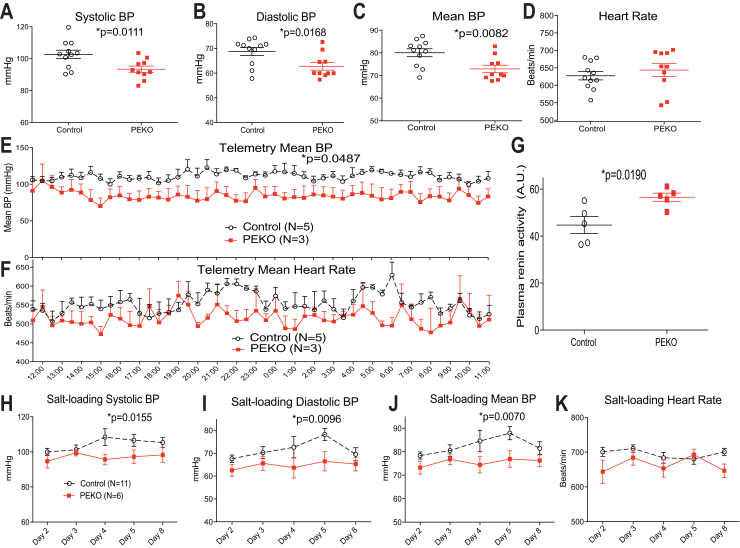
PEKO mice have decreased blood pressure and increased plasma renin activity. A–D: Tail cuff determinations of systolic (A), diastolic (B), and mean (C) blood pressure as well as heart rate (D) in control and PEKO mice. E and F: Telemetry-determined blood pressure and heart rate in unrestrained mice. G: Plasma renin activity in control and PEKO mice. H–K: Tail cuff determinations of systolic (H), diastolic (I), and mean (J) blood pressure as well as heart rate (K) in control and PEKO mice in the setting of salt loading. Data are presented as individual data points with mean ± SEM or mean ± SEM over time. PEKO, PexRAP endothelial knockout.

Systolic, diastolic, and mean blood pressure determined by tail cuff were lower in PEKO as compared with littermate controls without affecting heart rate (Fig. 2A–D). Lower blood pressure without a compensatory increase in heart rate was confirmed using telemetry sensors in unrestrained animals after recovery from surgery (Fig. 2E, F). Plasma renin activity was increased in PEKO mice (Fig. 2G), reflecting an appropriate activation of the renin-angiotensin system in response to decreased blood pressure. Feeding a high-salt diet for 8 days increased blood pressure in control but not PEKO mice (Fig. 2H–K). To demonstrate tamoxifen-dependence, a separate cohort of PexRAP^lox/lox^ mice without Cre and PexRAP^lox/lox^ mice with Cre were treated with corn oil (the vehicle for tamoxifen) for 5 consecutive days at age 35 days, then the blood pressures were measured 1 month later. There was no significant difference in blood pressure between control and PEKO mice not given tamoxifen (supplemental Fig. S4A–D). Conventional whole body knockout of PexRAP induces embryonic lethality, but PexRAP heterozygotes are viable (31). Mice with global heterozygosity for PexRAP deficiency had the same blood pressure as littermate controls (supplemental Fig. S4E–H).

**Fig. 3:**
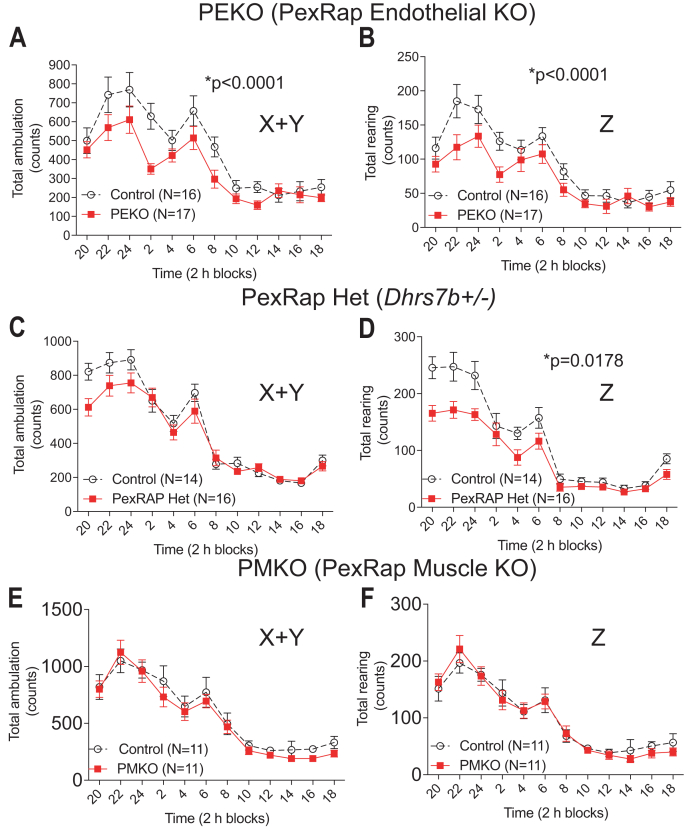
Decreased physical activity in PEKO mice. A and B: Home cage movement in the horizontal plane (X+Y) (A) and vertical rearing (Z) (B) in control mice and mice with endothelial-specific PexRAP deficiency (PEKO mice). C, D, Home cage movement in the horizontal plane (C) and vertical rearing (D) in control mice and mice with whole body heterozygous PexRAP deficiency (*Dhrs7b*+/- mice). E and F: Home cage movement in the horizontal plane (E) and vertical rearing (F) in control mice and mice with skeletal muscle-specific PexRAP deficiency (PMKO mice). Data are presented as mean ± SEM for the preceding 2 h time period. PEKO, PexRAP endothelial knockout; PMKO, PexRAP muscle knockout.

PEKO mice with inducible endothelial PexRAP deficiency showed visual responses comparable to controls using full-field flash electroretinography under photopic and scotopic conditions (supplemental Fig. S5A), indicating that PEKO animals are not blind. Forelimb strength measurements of PEKO and control mice were comparable (supplemental Fig. S5B). However, PEKO mice moved less, with decreased ambulation (X+Y axis) and rearing (Z axis) when monitored by beam breaks in home cages over 24 h (Fig. 3A, B). Heterozygous PexRAP animals, without a blood pressure phenotype (supplemental Fig. S4), also moved less (Fig. 3D). To determine whether PexRAP deficiency in a tissue relevant to physical activity affected ambulation, we generated PMKO mice in the C57BL/6J background using Cre driven by human α-skeletal actin. PexRAP expression was decreased in skeletal muscles but not liver of PMKO mice (supplemental Fig. S5C–E). Despite skeletal muscle deficiency of PexRAP in PMKO mice, ambulation and rearing were the same in PMKO and littermate control mice (Fig. 3E, F), suggesting that the loss of PexRAP at the endothelium has a relatively specific effect on behavior.

**Fig. 4:**
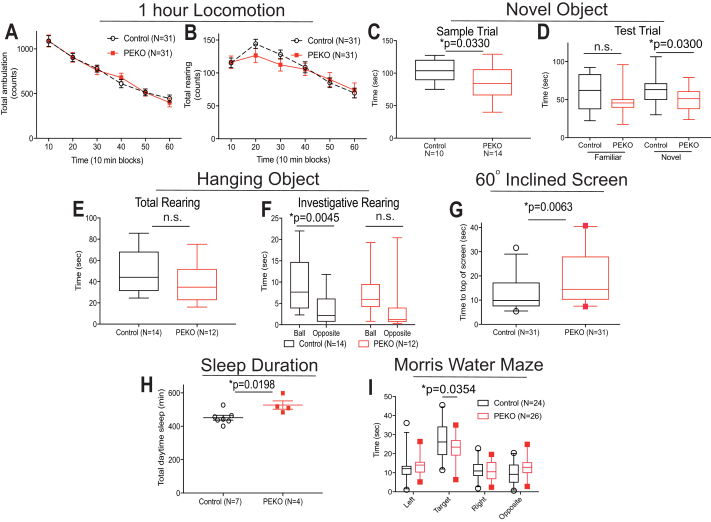
Behavioral phenotyping in PEKO and control mice. A and B: One hour locomotor activity testing for ambulatory activity (A) and vertical rearing (B) performed with lights on during daytime in control and PEKO mice. C and D: Novel object recognition testing employing a sample trial (C) and test trial (D). E and F: Assessment of attention devoted to a hanging object included measurements of total rearing time as a control procedure (E) and investigative rearing time as an index of attention (F). G: Time required for control and PEKO mice to reverse position and climb to the top of a 60° inclined screen. H: Daytime sleep duration. I: Probe trial data from the Morris water maze procedure. Data are presented as mean ± SEM for the preceding 10 min time period, box and whisker plots, or individual data points with mean ± SEM. PEKO, PexRAP endothelial knockout.

PEKO and control mice showed no differences in ambulation or vertical rearing during 1 h locomotor activity testing (Fig. 4A, B), conducted with lights on during daytime, consistent with similar capacity for movement in a novel testing environment. In novel object recognition testing (based mostly on tactile cues), PEKO as compared with control mice spent less time investigating the same object pairs during the sample trial and less time investigating the novel object but not the familiar object during the test trial (Fig. 4C, D). These results suggest limited awareness of objects and environmental changes, which might impair retention of experiences. PEKO investigative time for a novel hanging object (ball) did not differ from investigative time for an empty space (no object), whereas control mice spent more rearing time investigating the hanging ball as compared with the empty space (Fig. 4E, F). These results are consistent with the findings of novel object recognition testing because PEKO mice show little interest in environmental events. PEKO mice required more time to climb to the top of a 60° inclined screen (Fig. 4G). This slow movement and greater daytime sleep duration in PEKO mice (Fig. 4H) suggest decreased arousal. In Morris water maze testing, PEKO and control mice had comparable responses in cued and place trials (supplemental Fig. S6A–F), consistent with learning. However, PEKO animals spent significantly less time in the target quadrant during the probe trial (Fig. 4I), suggesting a deficit in spatial memory for PEKO mice.

Behavioral phenotyping included males and females for both genotypes and no differences were detected between sexes.

### Neurodegeneration in PEKO mice

Selected regions of PEKO brain appeared normal by Nissl staining (supplemental Fig. S7A, B), and there were no significant differences in brain weights at 6 months of age (supplemental Fig. S7C, D). To screen for major effects on blood-brain barrier function, IgG staining was performed (supplemental Fig. S7E) on three animals per genotype, and no IgG halos associated with microvessels were detected in PEKO mice, suggesting that the blood-brain barrier was intact for large molecules.

**Fig. 5:**
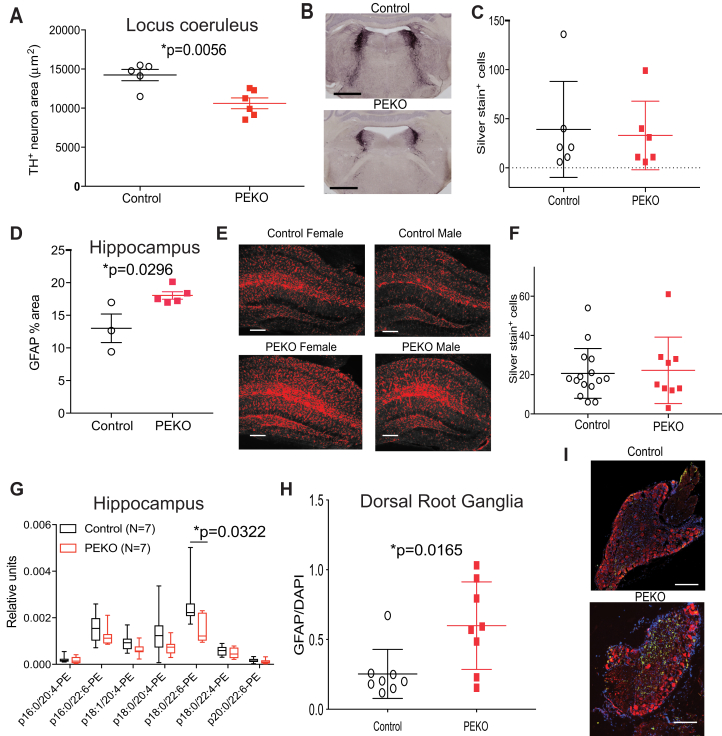
Characterization of the locus coeruleus, hippocampus, and dorsal root ganglia in PEKO and control mice. A: Area of tyrosine hydroxylase positive neurons based on genotype. B: Representative images from sections quantified for panel A. Scale = 1 mm. C: Locus coeruleus silver staining. D: GFAP quantification in hippocampus based on genotype. E: Representative images (red = GFAP) from sections quantified for panel D. Scale = 100 microns. F: Hippocampal silver staining. G: Quantification of PE plasmalogens in the hippocampus of control and PEKO mice. H: GFAP quantification in dorsal root ganglia based on genotype. I: Representative images (green = GFAP, blue = DAPI, red = TuJ1) from sections quantified for panel H. Scale = 100 microns. Data are presented as individual data points with mean ± SEM or box and whisker plots. PE, phosphatidylethanolamine; PEKO, PexRAP endothelial knockout.

Because PEKO mice have evidence of autonomic dysfunction, impaired arousal, and memory deficits, we characterized the locus coeruleus (LC) and hippocampus. Tyrosine hydroxylase-positive neuron area was decreased in PEKO compared with control LC with no difference in silver staining (Fig. 5A–C). Gliosis was increased in PEKO compared with control hippocampus (red = GFAP) with no difference in silver staining (Fig. 5D–F). The PE plasmalogen 18:0/22:6 has been reported to increase in the hippocampus of C57BL/6J mice during the acquisition of spatial memory associated with Morris water maze training (51). The content of this species was decreased in the hippocampus of PEKO as compared with control mice (Fig. 5G). Plasmalogens are required for normal peripheral nervous system function (52). Gliosis was increased in uninjured dorsal root ganglia of PEKO as compared with control mice (Fig. 5H, I; green = GFAP, blue = DAPI, red = TuJ1), suggesting that ether lipids derived from the endothelium (as opposed to those generated by neurons) can affect the peripheral as well as the central nervous system.

### Intact cortical functional connectivity in PEKO mice despite blood pressure phenotype

**Fig. 6:**
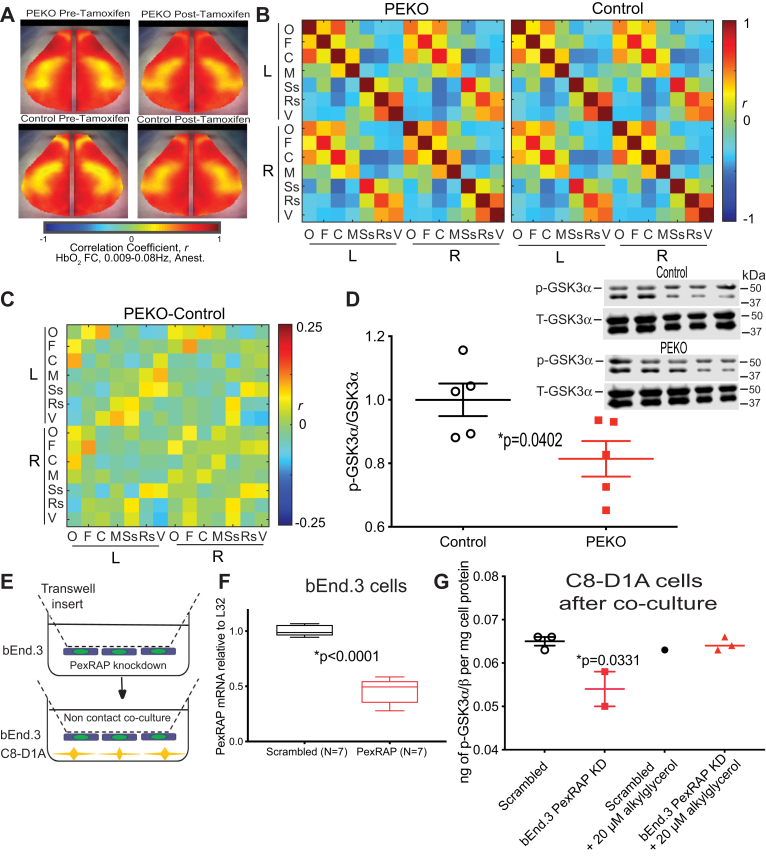
Functional connectivity and co-culture rescue of altered GSK phosphorylation. A: Bilateral functional connectivity maps for control and PEKO mice pretamoxifen and posttamoxifen injection. B: Functional connectivity matrices for PEKO and control mice posttamoxifen treatment using seven canonical seeds: O = olfactory, F = frontal, C = cingulate, M = motor, Ss = somatosensory, Rs = retrosplenial, and V = visual. C: Differences between PEKO and control matrices. D: GSK3α phosphorylation by Western blotting in control and PEKO cortex from female mice (representative Western blots presented in inset; p = phosphorylated, T = total). E: Co-culture strategy. F: PexRAP mRNA in bEnd.3 cells. G: GSK3 phosphorylation in C8-D1A cells after co-culture with bEnd.3 PexRAP knockdown cells and after co-culture in the presence of the plasmalogen precursor alkylglycerol. ∗*P* = 0.0331 by ANOVA. GSK3, glycogen synthase kinase-3; PEKO, PexRAP endothelial knockout.

Because the LC (53) and hippocampus (54) interact with the cerebral cortex, we mapped cortical functional connectivity in mice using optical intrinsic signal imaging, which uses tissue spectroscopy to quantify neurovascular responses based on changes in oxyhemoglobin and deoxyhemoglobin (44). PEKO mice have decreased blood pressure, which can cause ischemia, and cortical functional connectivity is substantially disrupted in mouse models of ischemic stroke (55). Despite their blood pressure phenotype after tamoxifen, PEKO mice have normal homotopic functional connectivity (before and after tamoxifen) with subtle differences compared with WT (Fig. 6A). Posttamoxifen functional connectivity matrices were calculated (Fig. 6B) using canonical seeds (O = olfactory, F = frontal, C = cingulate, M = motor, Ss = somatosensory, Rs = retrosplenial, V = visual), and differences between PEKO and WT matrices were determined (Fig. 6C). The cluster of differences in Fig. 6C suggest physiologically altered cortical connections, especially involving the retrosplenial and visual cortices. These altered connections were not significant after correction for multiple comparisons because of significantly increased variance (*P* = 0.0369) across seeds in PEKO mice (supplemental Fig. S8A, B).

Increased activity of GSK3 is associated with defective memory formation and neurodegeneration (56). GSK3 regulation involves phosphorylation, which inhibits enzyme activity. GSK3α phosphorylation was decreased in female PEKO cortex as compared with controls (Fig. 6D). To model the interaction between endothelial and neuronal cells, we performed noncontact co-culture experiments using mouse endothelium-like bEnd.3 cells (57) and mouse astrocyte-like C8-D1A cells (58) (Fig. 6E). bEnd.3 cells were treated with scrambled or mouse PexRAP-specific shRNA virus, then transferred to co-culture wells containing C8-D1A cells. PexRAP expression was decreased in knockdown as compared with control bEND.3 cells (Fig. 6F). In a proteomic analysis, eight proteins were increased by >10-fold in C8-D1A cells co-cultured with endothelial cells with PexRAP deficiency (supplemental Table S1). These included glial fibrillary acidic protein (Gfap), which is increased in hippocampus and dorsal root ganglia of PEKO mice (Fig. 5), and three proteins implicated in neurodegeneration, Uba1Y, Ubql2, and Gstm2.

Phosphorylated GSK3 was decreased in C8-D1A cells co-cultured with PexRAP-deficient bEnd.3 cells for 24 h (Fig. 6G). Figure 6G shows a single experiment; similar results were seen in four separate experiments, three with alkylglycerol rescue. Symbols in Fig. 6G represent independent cultures (biological replicates), and each symbol is the mean of multiple technical replicates performed on each culture. In four experiments, co-culture with PexRAP-deficient bEnd.3 cells decreased phosphorylated GSK3 by 17%, 15%, 20%, and 28% in C8-D1A cells. Alkylglycerol is an ether lipid that crosses plasma membranes and can be utilized for synthesis of complex phospholipids such as plasmalogens. Including alkylglycerol in the co-culture rescued decreased GSK3 phosphorylation in C8-D1A cells (Fig. 6G). Alkylglycerol has access to both endothelial cells (bEnd.3) and glial cells (C8-D1A) under these conditions. When only C8-D1A cells were cultured for 24 h in the presence of alkylglycerol (AG, 20 μM 16:0 alkylglycerol and 18:0 alkylglycerol), phosphorylated GSK was decreased (supplemental Fig. S9A). Alkylglycerol incubation increases p-GSK in C8-D1A cells when co-cultured with PexRAP-deficient bEnd.3 cells (Fig. 6G) but decreases pGSK in C8-D1A cells alone, suggesting that the rescue of decreased GSK3 phosphorylation with co-culture requires the presence of the endothelial cells.

In adipose tissue, PexRAP is found in the nucleus where it disrupts PRDM16-mediated gene expression (31). This raises the possibility that processes beyond ether lipid synthesis could be responsible for the findings in PEKO mice. However, subcellular fractionation of bEnd.3 cells and stromal vascular cells from brown adipose tissue indicates that PexRAP has limited access to the nucleus in endothelial cells (supplemental Fig. S9B).

## Discussion

Gray matter and white matter in human brain are ∼40–80% lipid (59). Plasmalogens, unique lipids that are most abundant in neural tissues, are characterized by a vinyl ether bond (60) that confers plasma membrane properties essential for normal neuronal function (17, 61). The enzymes required to synthesize ether lipids include PexRAP, a peroxisomal protein implicated in thermogenesis and insulin sensitivity (31, 62). Because blood vessels are intimately related to neurodegeneration and endothelial dysfunction characterizes hypertension as well as aging, we induced inactivation of PexRAP in endothelial cells of adult mice.

Surprisingly, endothelial-specific deficiency of PexRAP in adult mice decreased circulating plasmalogens. Systemic plasmalogen metabolism is poorly understood, but these complex lipids are present in nearly all foods and probably synthesized by all tissues. Plasmalogens are thought to reach the circulation in liver-generated lipoproteins (63), but conventional plasma lipids as well as diacyl phospholipids, alkyl PC species (nonplasmalogen ether lipids), sphingomyelins, and PIs were unaffected in PEKO mice. These findings suggest that endothelial cells, which line all blood vessels, weigh ∼1 kg in humans (64), and have an estimated surface area of ∼20 m^2^ in the human brain (65), may contribute to the circulating pool of PE plasmalogens.

Plasmalogens are labile in gray matter with a half-life of less than 25 min (66) and stable in myelin with a half-life of weeks to months (67), consistent with the notion that these lipids serve a metabolic function in neurons and a structural function in myelin. If plasmalogens (or their precursors) generated by the endothelium maintain metabolic signaling in neurons, the loss of this pool would be predicted to cause neurodegeneration. PE plasmalogens are decreased in the plasma of PEKO mice, but it is likely the lack of localized production of these lipids in close proximity to neural tissue that results in the phenotype of PEKO mice.

Inactivating endothelial ether lipid synthesis in PEKO mice lowered systolic and diastolic blood pressure and appropriately activated the renin angiotensin system. PEKO mice had a normal response to hindlimb ischemia, strongly suggesting that the loss of endothelial lipid synthesis does not impair the ability of the PEKO vasculature to regulate the complex cascade of oxygenation, metabolism, angiogenesis, and perfusion required for vascular health. However, PEKO mice were unresponsive to salt-loading, an activator of the sympathetic nervous system, and demonstrated loss of tyrosine hydroxylase staining neurons in the LC, a major mediator of autonomic function and arousal (68). PEKO mice also showed diminished movement and rearing, increased sleep, and impaired attention as well as mild retention deficits in the setting of hippocampal gliosis.

The LC is involved in blood pressure, attention, and memory. Stimulation of the LC elevates blood pressure (69, 70, 71), and this brainstem nucleus participates in salt-induced hypertension (72). The wakefulness-promoting function of the LC is related in part to extensive projections to the cortex (68), and LC activity has been implicated in cortical-dependent and hippocampal-dependent attention and memory (73). Anatomical connectivity exists between the LC and hippocampus and optogenetic inhibition of LC signaling impairs memory (74). Neuronal loss in the human LC may exceed degeneration of the nucleus basalis in Alzheimer's disease and the substantia nigra in Parkinson's disease (75).

Plasmalogen deficiency in mice (52) is associated with activation (decreased phosphorylation) of GSK3, which occurs in neurodegeneration and impaired memory. Female PEKO mice had altered GSK3 phosphorylation in the cortex, co-culture of PexRAP-deficient endothelium-like cells with WT astrocyte-like cells altered GSK3 phosphorylation in the astrocyte-like cells, and this effect was rescued by the ether lipid precursor alkylglycerol. The add-back data do not distinguish between an effect mediated by plasmalogens as opposed to alkyl ether lipids, but findings in mice and cells suggest that disruption of PexRAP in endothelial cells decreases plasmalogens that normally have trophic effects on neural tissue. There is precedent for endothelial-derived factors affecting the function of neurons and glia (76, 77).

Our results suggest an intimate relationship between the generation of plasmalogens by brain endothelial cells and neural tissue. Individual neurons are usually closer to a brain capillary than a CSF compartment (65), making it likely that the heterogeneous brain capillary endothelium (78) influences the neural microenvironment. The mechanism by which endothelial plasmalogens impact neurons is not clear. One possibility is that failure of delivery of endothelial cell plasmalogens alters neuron membrane architecture to disrupt intracellular signaling. Ether lipid metabolites act as precursors for glycosyl-phosphatidyl-inositol anchors (18), important for cell signaling. In the peripheral nervous system, plasmalogens are required for normal AKT signaling and Schwann cell differentiation (52). Plasmalogen deficiency in the peripheral nervous system activates an isoform of GSK3, and GSK3 inhibition restores Schwann cell defects. Inhibiting GSK3 in PEKO mice followed by characterizing blood pressure and behavior phenotypes represents an approach for future studies.

Global PexRAP (*Dhrs7b*) deficiency in mice is lethal (31). The human gene is located in a region prone to rearrangements associated with the Smith-Magenis and Potocki-Lupksi syndromes characterized by cognitive impairment and adverse effects on multiple systems (79). PexRAP interacts with oligomeric β-amyloid (80), its gene expression decreases with age in humans (81), and peroxisomal proteins decrease with age in *C. elegans* (82).

Peroxisomes are responsible for synthesis of ethanolamine plasmalogens through a single nonredundant pathway that is apparently ubiquitous and functional in brain (83). Neurodegeneration following endothelial-specific PexRAP inactivation suggests that neurons and glia behind the blood-brain barrier preferentially derive plasmalogens from the cells that form this barrier, cerebrovascular endothelial cells. This process may involve Mfsd2a, a blood-brain barrier transporter for lysophosphatidylcholine that also transports lysophosphatidylethanolamine and lysoplasmalogen (84).

Our findings suggest that disrupting production of plasmalogens by the endothelium, perhaps as a consequence of hypertension, diabetes, salt intake (9), and other vascular risk factors, may lead to neurodegeneration that is associated with decreased blood pressure in later life.

## Data availability

Data are contained within the paper with the exception of mass spectrometry proteomics data that have been deposited to the ProteomeXchange Consortium (http://proteomecentral.proteomexchange.org/) via the PRIDE partner repository with the data set identifier PXD024116.

## Supplemental data

This article contains supplemental data.

## Conflict of interest

The authors declare that they have no conflicts of interest relevant to this manuscript.
