# Supplementary material for: Endothelial ether lipids link the vasculature to blood pressure, behavior, and neurodegeneration
Source: J Lipid Res. 2021 Apr 21;62:100079. doi: 10.1016/j.jlr.2021.100079 (PMC8144742; doi:10.1016/j.jlr.2021.100079)
Supplement: Supplemental Figs. S1 to S9 and Supplemental Table S1 [file mmc1.pdf]

SUPPLEMENTAL INFORMATION:

Endothelial ether lipids link the vasculature to blood pressure, behavior, and neurodegeneration

Larry D. Spears[1], Sangeeta Adak[1], Guifang Dong[1,2], Xiaochao Wei[1], George Spyropoulos[3], Qiang Zhang[1], Li Yin[1], Chu Feng[1], Donghua Hu[1], Irfan J. Lodhi[1], Fong-Fu Hsu[1], Rithwick Rajagopal[4], Kevin K. Noguchi[5], Carmen M. Halabi[3], Lindsey Brier[6], Annie R. Bice[6], Brian V. Lananna[7], Erik S. Musiek[7], Oshri Avraham[8], Valeria Cavalli[8], Jerrah K. Holth[7], David M. Holtzman[7], David F. Wozniak[5], Joseph P. Culver[6], and Clay F. Semenkovich[1,9]

[1]Division of Endocrinology, Metabolism & Lipid Research, Department of Medicine, Washington University, St. Louis, MO 63110, USA, [2]Hubei Key Laboratory of Animal Nutrition and Feed Science, Wuhan Polytechnic University, Wuhan 430023, China, and Departments of [3]Pediatrics, [4]Ophthalmology & Visual Sciences, [5]Psychiatry, [6]Radiology, [7]Neurology, [8]Neuroscience, and [9]Cell Biology & Physiology, Washington University, St. Louis, MO 63110, USA

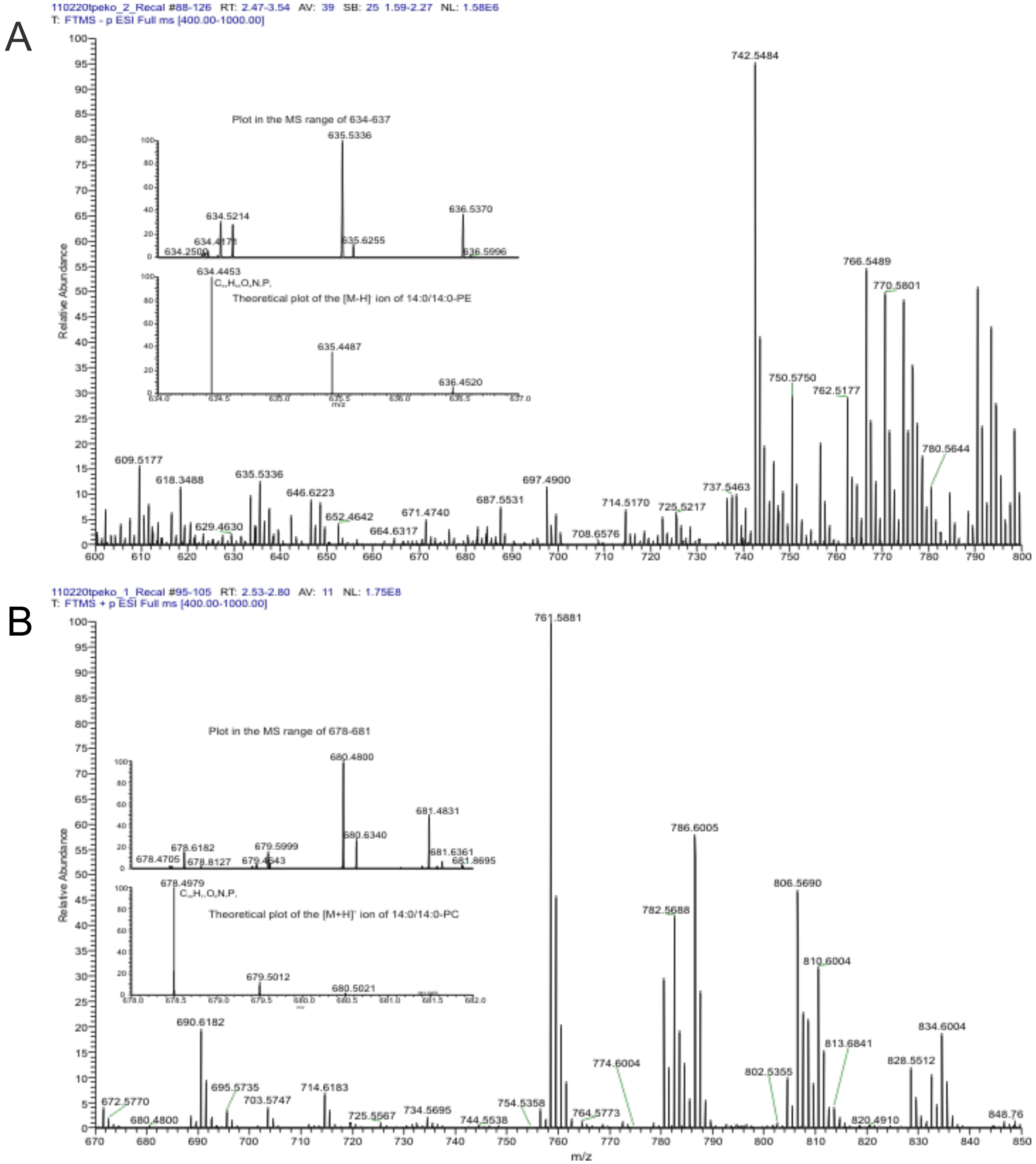

Supplemental Fig. S1. Validation of internal standards in PEKO mice. Lipid extracts analyzed from control and PEKO mice without adding 14:0-14:0 diradyl glycerophospholipids as internal standards showed that these internal standards are absent in these samples. Results for PEKO samples are presented above. Panel A is the high resolution negative-ion ESI MS of the lipid extract to detect PE as the $[M-H]^-$ ions. For the inset, top is the plot of the m/z 634-637, bottom is the theoretical plot of the $[M-H]^-$ ion of 14:0-14:0)-PE (elemental composition: $C_{33}H_{65}O_8NP^-$; 634.4453 Da). No ion in the top plot matches both the masses and the pattern of the bottom theoretical plot. Panel B presents PC species in the same sample obtained as the $[M+H]^+$ ions in the positive ion mode. For the inset, no ion in the top plot in the m/z 678-681 region matches the bottom theoretical plot of the $[M+H]^+$ ion of 14:0-14:0)-PC (elemental composition: $C_{33}H_{71}O_8NP^+$; 678.4979 Da). Note that the abundances of these background ions (same nominal mass, but different species) are extremely low.

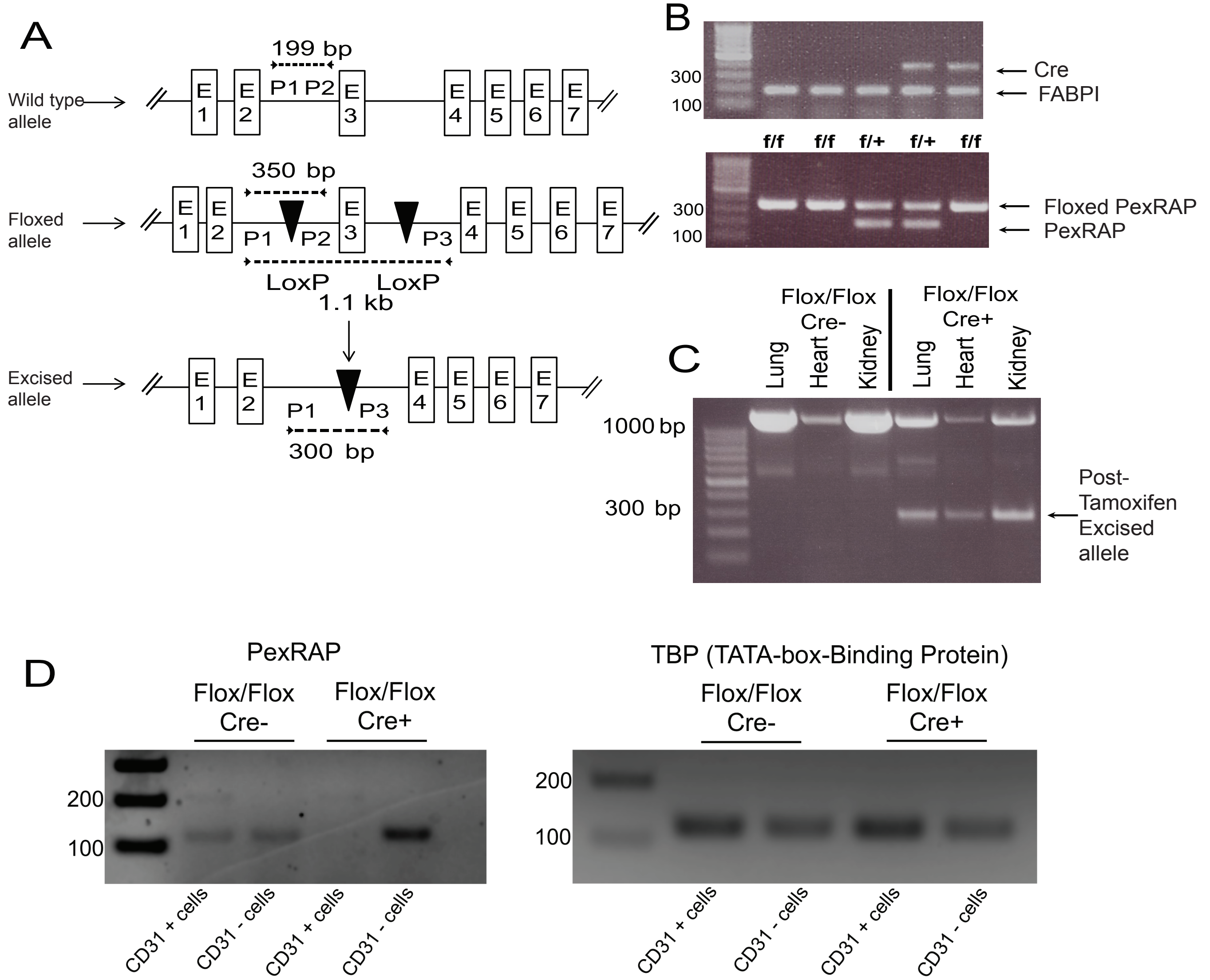

Supplemental Fig. S2. Characterization of PEKO mice. (A) Strategy for inactivating PexRAP. (B) Representative genotyping images for two Cre positive mice (top), and three mice with homozygous floxed alleles (f/f) and two mice with heterozygous floxed alleles (f/+) (bottom). (C) Genotyping of a mouse with floxed alleles treated with tamoxifen in the absence of Cre (left) and a mouse treated with tamoxifen in the presence of Cre leading to excision of exon 3 (right, indicated by the presence of the recombined 300 bp PCR product). (D) RT-PCR of PexRAP mRNA in isolated endothelial cells. CD31+ and CD31- cells were isolated from mouse lung using Dynabeads, followed by RT-PCR for PexRAP (forward ACTACTTTGGCCCTGTTGCT, reverse GATCGGAAAGGAATGCTGAT) and the control message TBP (forward AGAACAATCCAGACTAGCAGCA, reverse GGGAACTTCACATCACAGCTC). The absence of signal only in the CD31+ sample of the Cre+ mice is consistent with endothelial-specific inactivation.

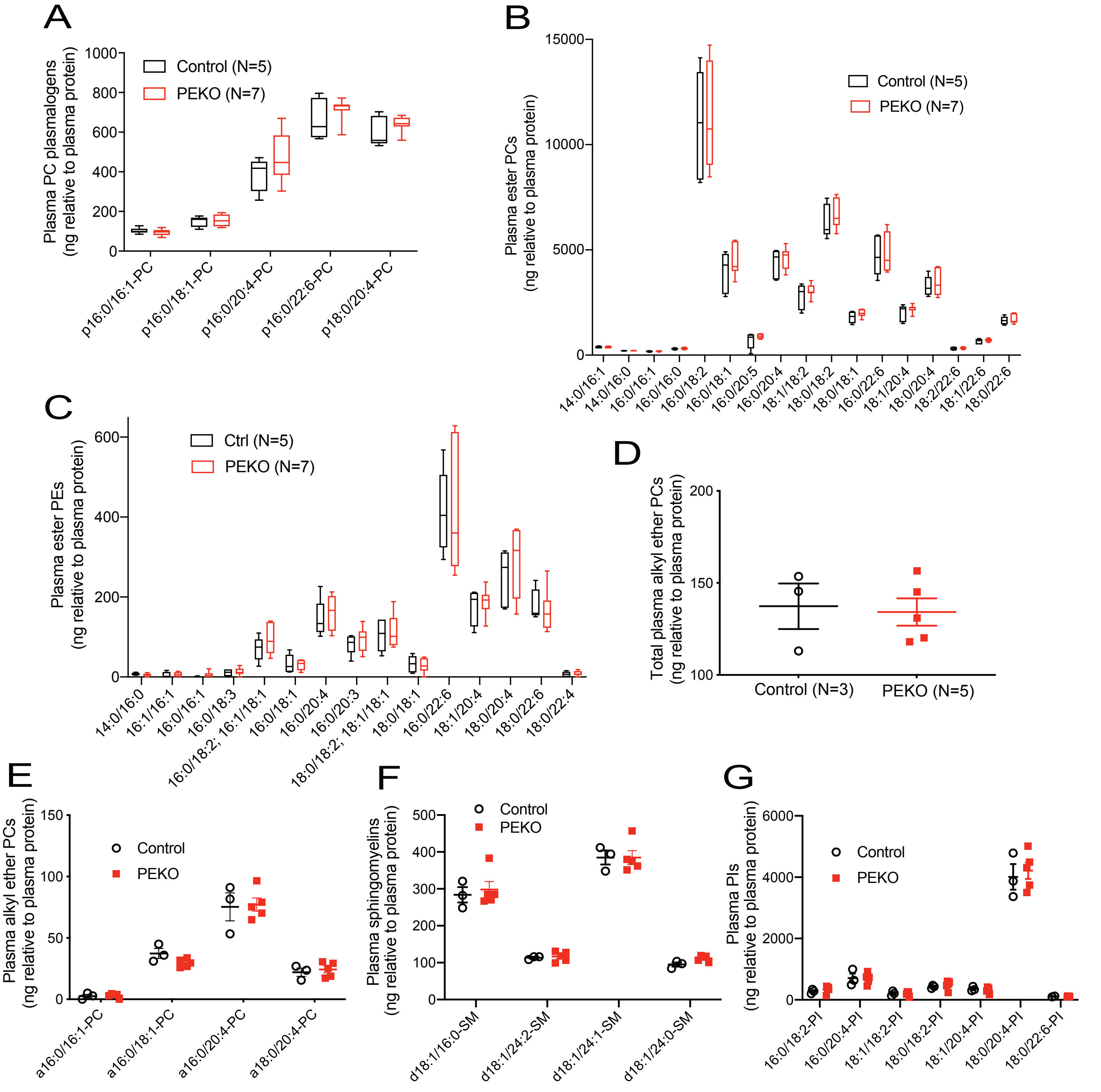

Supplemental Fig. S3. Lipidomics for PEKO and control mice. (A) Individual PC (phosphatidylcholine) plasmalogens determined by mass spectrometry in plasma from control and PEKO mice. Total PC plasmalogens are shown in Fig. 1D. (B) Individual ester PC species in plasma from control and PEKO mice. Total ester PCs are shown in Fig. 1E. (C) Individual ester PE species in plasma from control and PEKO mice. Total ester PEs are shown in Fig. 1F. (D) Total plasma alkyl ether PCs in control and PEKO mice. (E) Individual alkyl ether PCs quantified for Fig. S3D. (F) Individual plasma sphingomyelins in control and PEKO mice. (G) Individual PIs (phosphatidylinositol species) in control and PEKO mice.

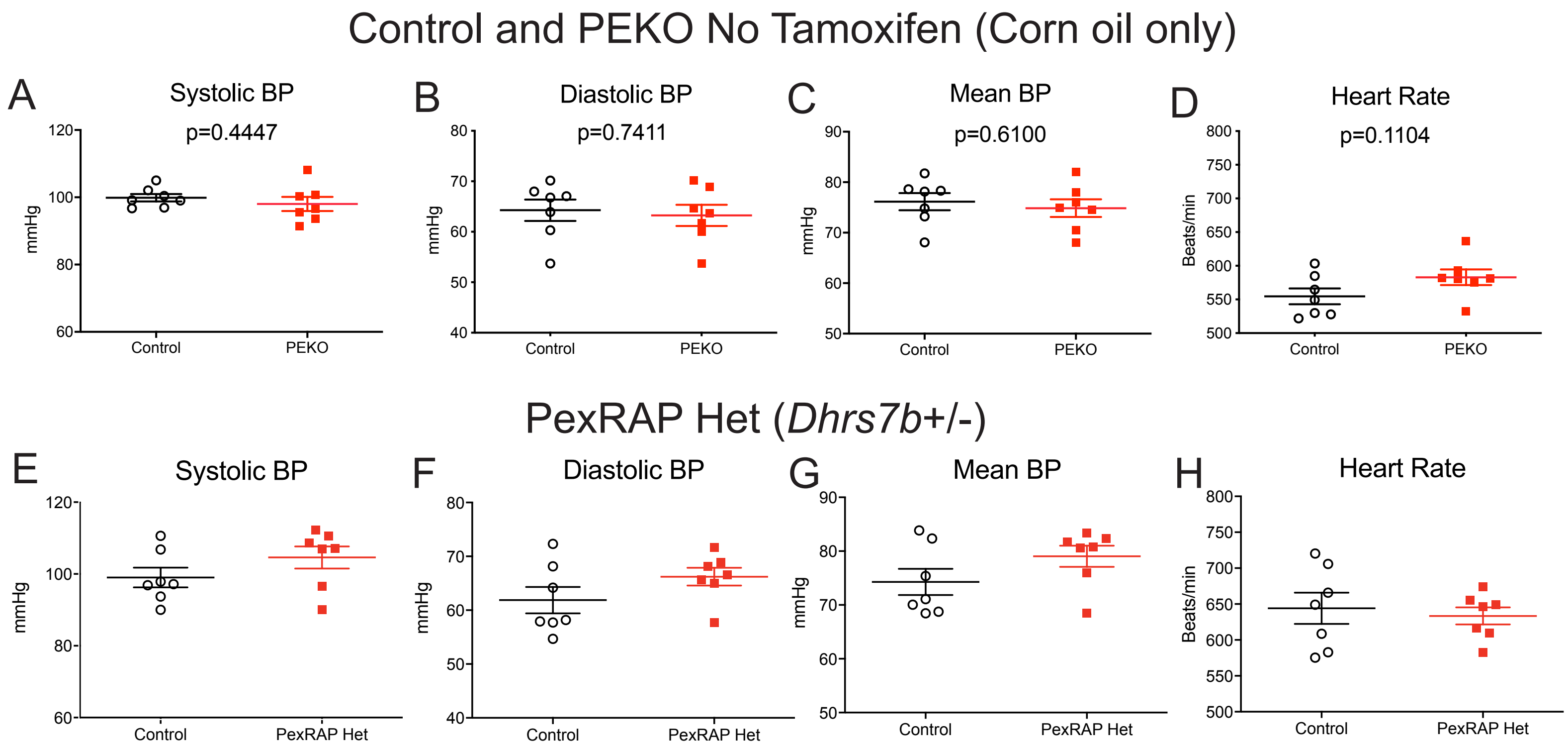

Supplemental Fig. S4. Blood pressure and heart rate in control and PEKO mice not treated with taxmoxifen (used to activate endothelial-specific Cre), and in whole body heterozygous PexRAP-deficient mice. (A,E) Systolic, (B,F) Diastolic, (C,G) Mean blood pressure and (D,H) Heart rate. For A-D, mice at the age of 35 days were subjected to five days of injection with corn oil only followed by analysis of blood pressure one month later, the same procedure followed for mice treated with tamoxifen shown in Fig. 2.

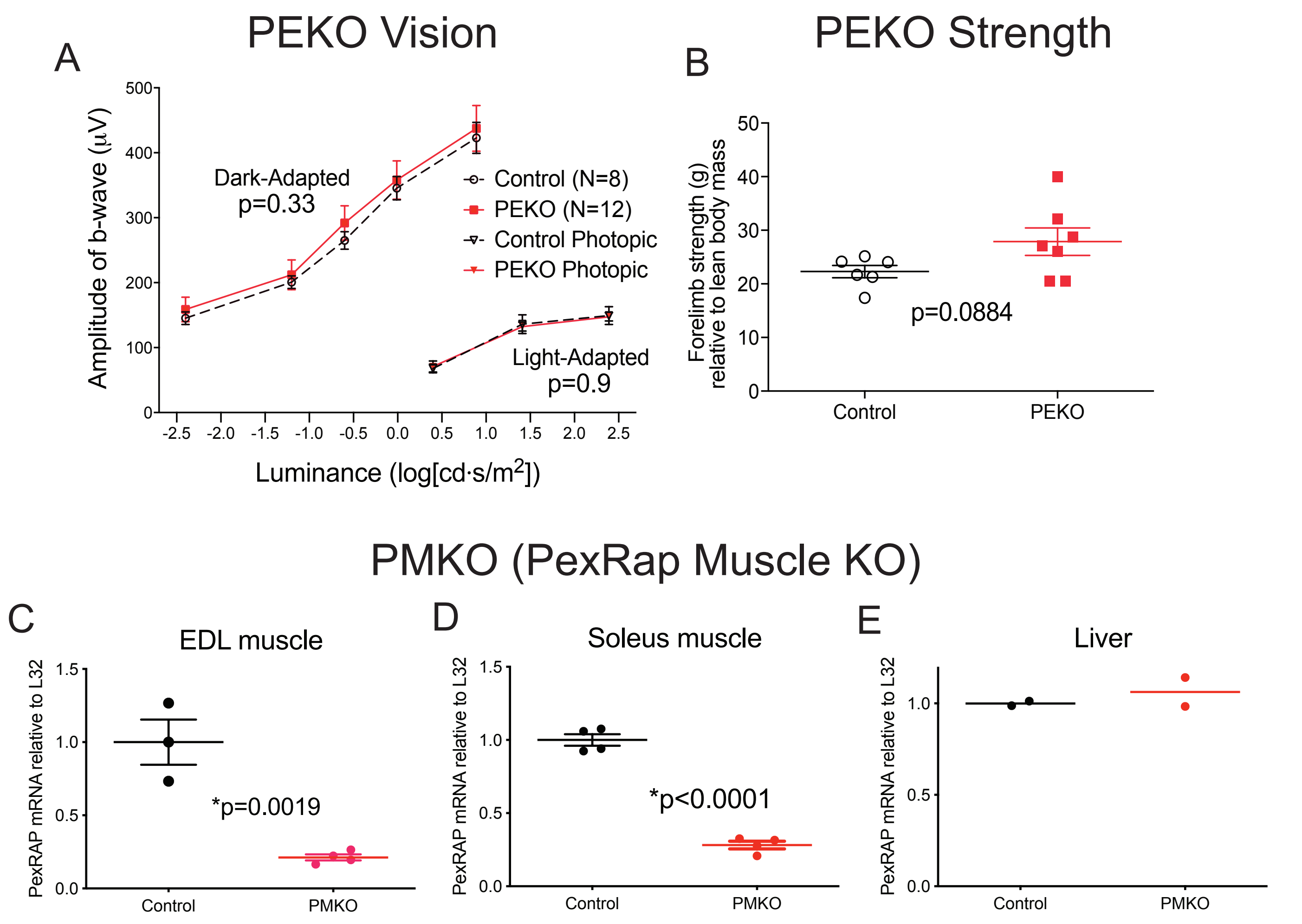

Supplemental Fig. S5. Characterization of PEKO and PMKO mice. (A) Electroretinography indicates that PEKO mice are not blind. (B) Forelimb strength is not decreased in PEKO mice. (C-E) Mice with muscle-specific PexRAP deficiency (PMKO mice) were assayed for PexRAP mRNA in EDL muscle (C), soleus muscle (D), and liver (E).

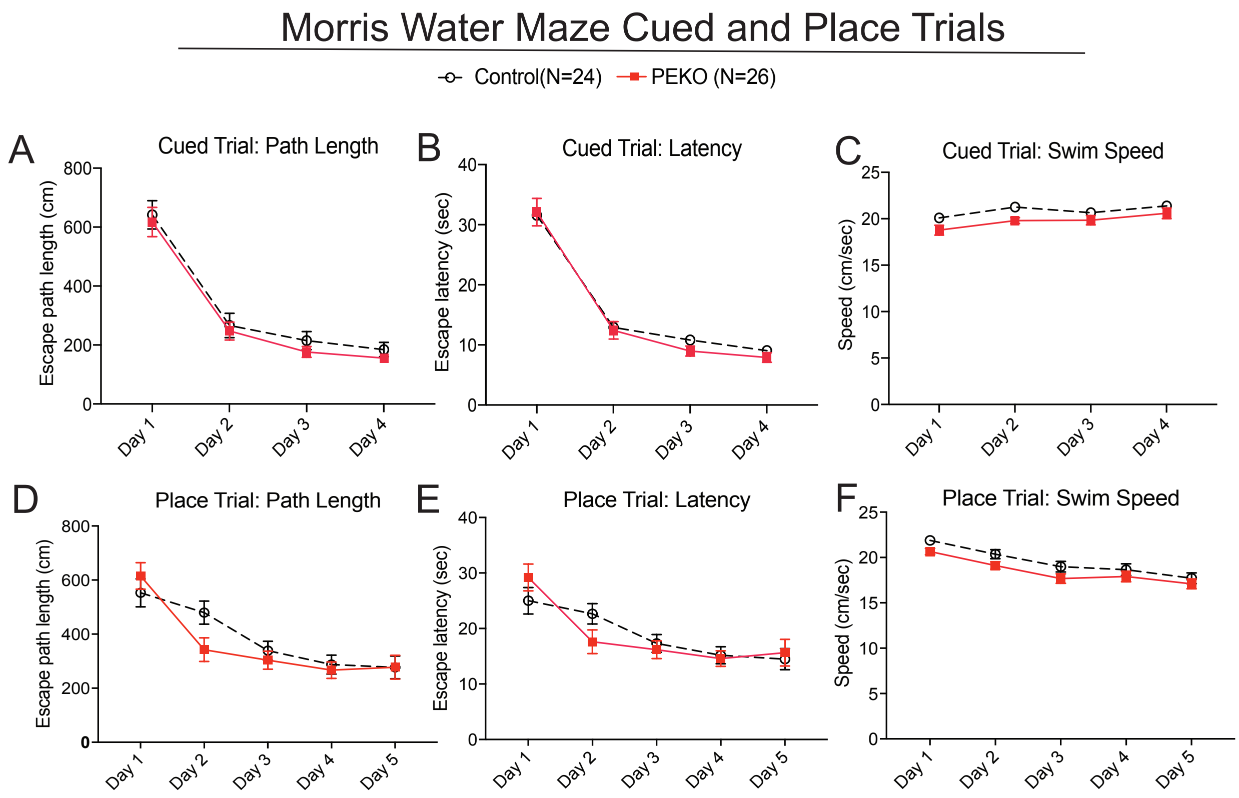

Supplemental Fig. S6. Morris water maze cued and place testing. Spatial learning and memory were assessed in separate cohorts of control and PEKO mice during cued (A-C) and place (D-F) trial components of the Morris water maze procedure.

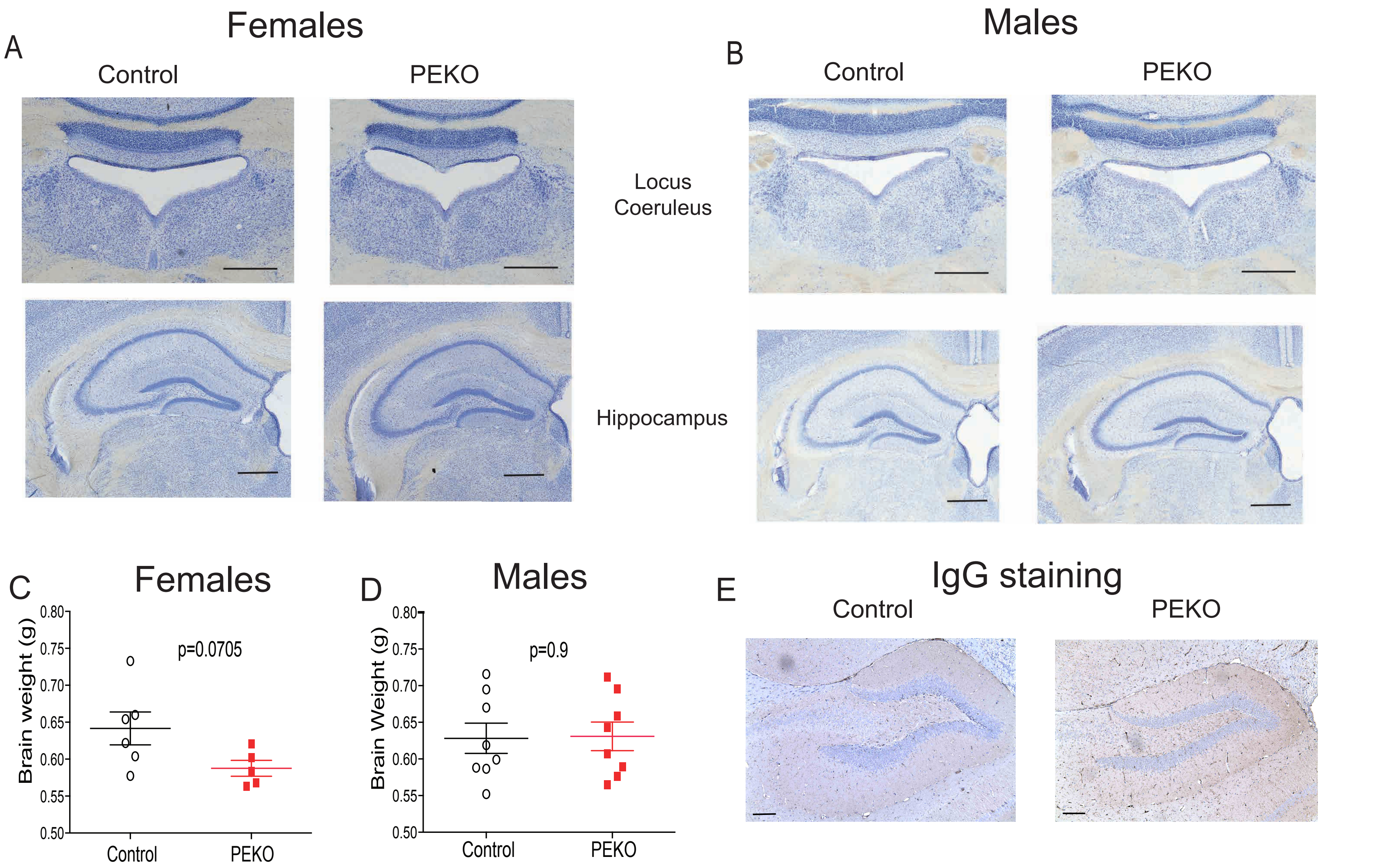

Supplemental Fig. S7. Characterization of mouse brain. Representative Nissl staining of female (A) and male (B) locus coeruleus and hippocampus for control and PEKO mice. Scale=500 microns. Multiple brains were examined for each genotype. Brain weights for female (C) and male (D) mice. (E) Representative IgG staining for control and PEKO mice. Scale=100 microns. Staining was performed in 3 control and 3 PEKO brains and compared to spleen as a positive control. Background staining was different for these photomicrographs but none of the brain images showed perivascular halos indicative of disruption of the blood brain barrier.

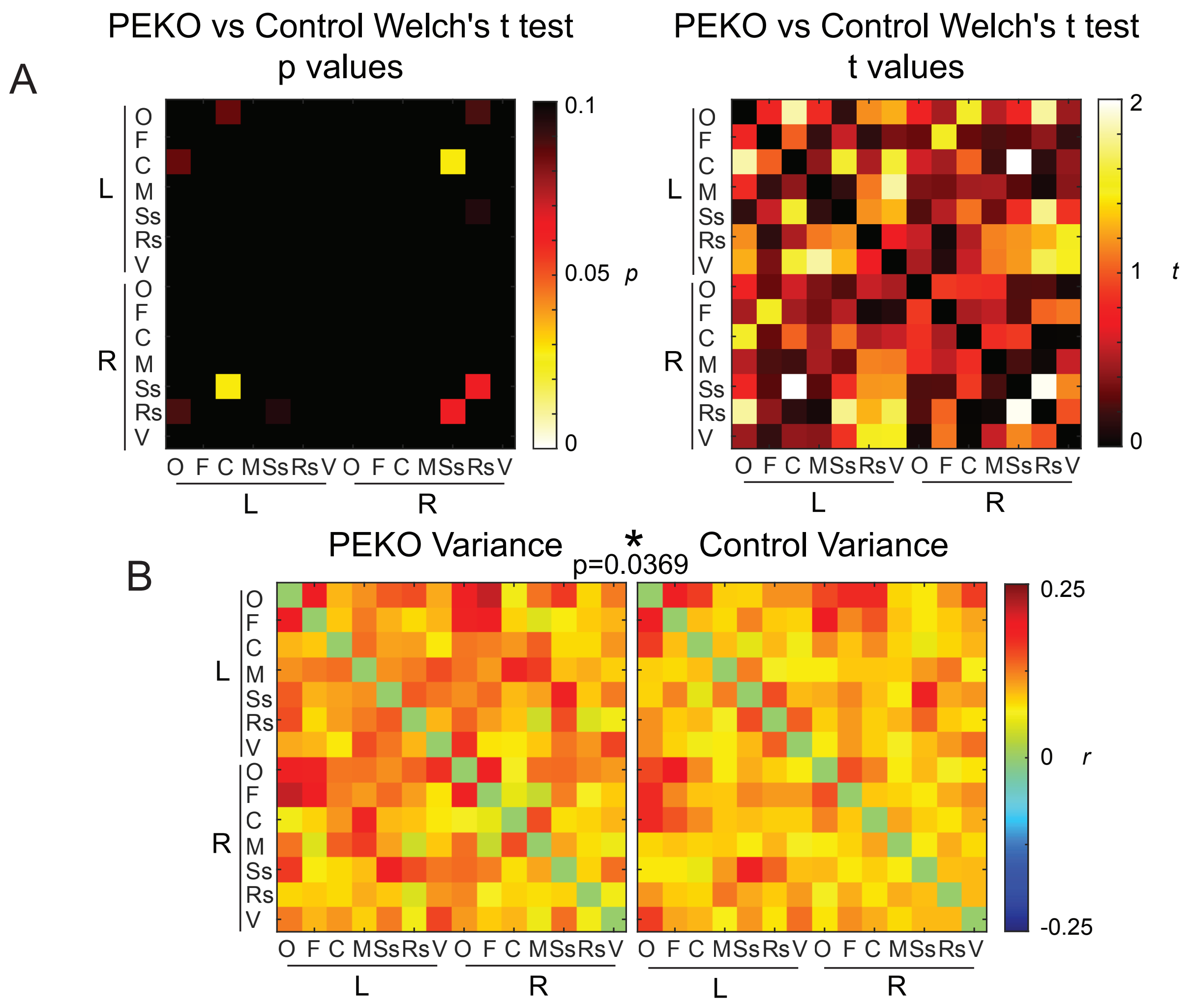

Supplemental Fig. S8. Analysis of variance in mouse cortical functional connectivity data. (A) Welch's t-test performed on functional connectivity mean values displayed in Figure 6 showed potentially altered cortical connections. The diagonal was blacked out and not compared. While underpowered, significance does not remain after Bonferroni correction for 84 comparisons. (B) Matrices displaying variance for each canonical seed connection analyzed in Figure 6. Variance across seeds in PEKO mice was significantly higher as compared to control mice (p=0.0369 by Welch's t-test).

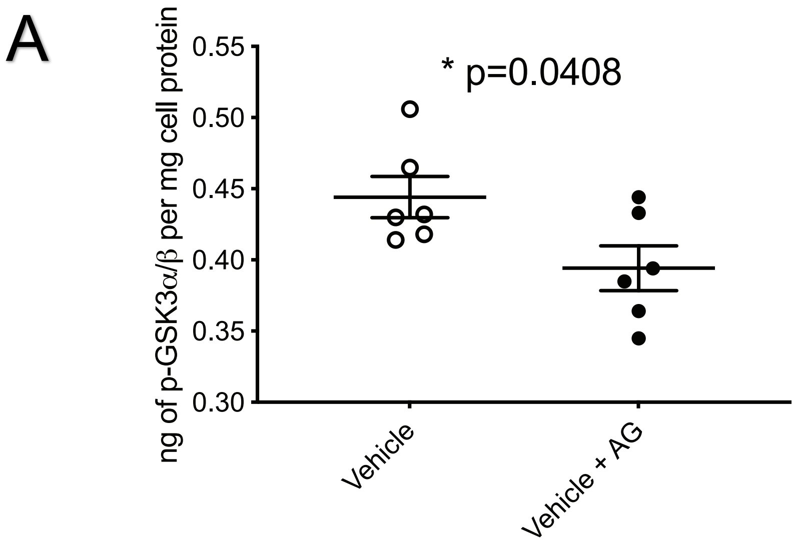

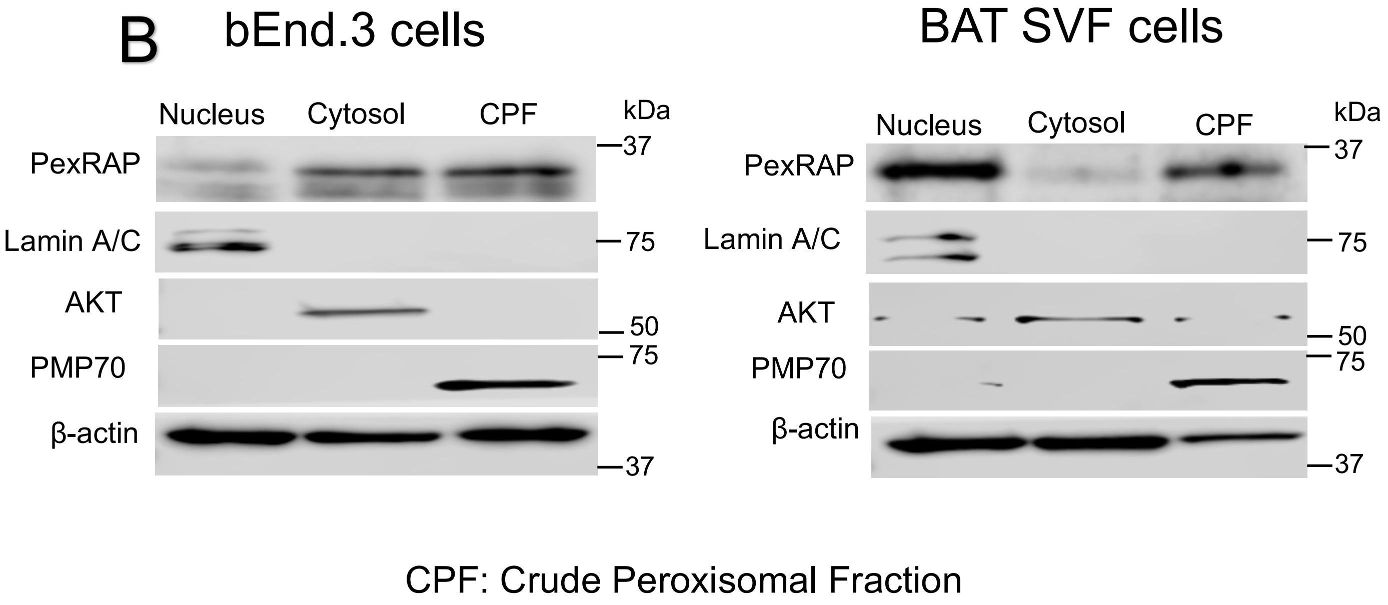

Supplemental Fig. S9. Effects of alkylglycerol on C8-D1A cells alone (A) and subcellular analysis of PexRAP (B). (A) C8-D1A astrocyte-like cells were cultured for 24 h in the absence or presence of 20 μM 1-O-hexadecyl-rac-glycerol (16:0 alkylglycerol) and 1-O-octadecyl-rac-glycerol (18:0 alkylglycerol) followed by determination of phosphorylated GSK. (B) Subcellular fractions of the nucleus, cytosol, and crude peroxisomes (CPF) were prepared from the brain endothelial cell line bEnd.3 and cells isolated from the stromal vascular fraction (SVF) of brown adipose tissue (BAT). Fractions were blotted for PexRAP as well as suitable organelle markers.

Fold increase in C8-D1A cells after co-culture with PexRAP-deficient bEnd.3 cells	Protein Identified	Accession Number	Molecular Weight	Unique Peptide Count	% Coverage
21.1	Glial fibrillary acidic protein OS=Mus musculus GN=Gfap PE=1 SV=4	sp|P03995|GFAP_MOUSE	54 kDa	135	0.59
18.0	Ubiquitin-like modifier-activating enzyme 1 Y OS=Mus musculus GN=Uba1y PE=2 SV=2	sp|P31254|UBA1Y_MOUSE	604 kDa	42	0.66
17.0	Beta-enolase OS=Mus musculus GN=Eno3 PE=1 SV=3	sp|P21550|ENOB_MOUSE	534 kDa	49	0.61
13.0	Talin-2 OS=Mus musculus GN=Tln2 PE=4 SV=1	tr|E9PUM4|E9PUM4_MOUSE	42 kDa	244	0.49
12.0	Ras GTPase-activating-like protein IQGAP2 OS=Mus musculus GN=Iqgap2 PE=1 SV=2	sp|Q3UQ44|IQGA2_MOUSE	226 kDa	56	0.71
10.0	60S ribosomal protein L35 OS=Mus musculus GN=Rpl35 PE=2 SV=1	sp|Q6ZWV7|RL35_MOUSE	72 kDa	60	0.89
10.0	Ubiquilin-2 OS=Mus musculus GN=Ubqln2 PE=1 SV=2	sp|Q9QZM0|UBQL2_MOUSE	74 kDa	237	0.53
10.0	Glutathione S-transferase Mu 2 OS=Mus musculus GN=Gstm2 PE=1 SV=2	sp|P15626|GSTM2_MOUSE	71 kDa	26	0.79

Supplemental Table S1. Proteins increased in C8-D1A cells following co-culture with PexRAP-deficient bEnd.3 cells. Of 2,384 proteins detected by mass spectrometry in astrocyte-like C8-D1A cells, eight increased by $\geq$10-fold when cells were co-cultured with PexRAP-deficient bEnd.3 cells as compared to cells co-cultured with PexRAP-replete bEnd.3 cells.
